# Metrological performances of the global chronic morbidity indicator of the Minimum European Health Module and implications for chronic disease prevalence and socioeconomic gradient estimations

**DOI:** 10.1093/eurpub/ckae064

**Published:** 2024-03-29

**Authors:** Joël Coste, Jean-Marie Robine, Herman Van Oyen, Laure Carcaillon-Bentata

**Affiliations:** French Public Health Agency (Santé Publique France), Saint-Maurice, France; MMDN, University of Montpellier, EPHE, Montpellier, France; PSL Research University, Paris, France; Department of Epidemiology and Public Health, Sciensano, Brussels, Belgium; Department of Public Health and Primary Care, Ghent University, Ghent, Belgium; French Public Health Agency (Santé Publique France), Saint-Maurice, France

## Abstract

**Background:**

Although the global chronic morbidity indicator (GCMI) of the Minimum European Health Module (MEHM) was not specifically designed to monitor chronic disease in the population, it is increasingly used for this purpose in Europe and elsewhere. However, its metrological characteristics have seldom been examined, with various sensitivity issues being raised. The present study investigated the metrological performances of the GCMI and analyzed its implications in terms of prevalence and demographic and socioeconomic gradients of chronic conditions in the population.

**Methods:**

We used data from two large French nationwide representative surveys with cross-sectional and longitudinal data conducted between 2010 and 2021. The surveys used MEHM and collected data on numerous chronic conditions and socioeconomic indicators. Criterion and predictive validity of the GCMI regarding chronic conditions and the resultant socioeconomic gradients were compared with indicators based on reports of individual chronic conditions.

**Results:**

GCMI sensitivity to capture chronic conditions varied from <20 to 80% depending on the chronic condition. Number of chronic conditions, gender, age and education were also associated with GCMI endorsement. However, the GCMI was predictive of mortality and activity limitations independently of individual conditions.

**Conclusion:**

The varying lack of sensitivity depending on the chronic condition and the respondent’s sociodemographic status may bias estimates of demographic and socioeconomic gradients compared with indicators based on reports of individual chronic conditions. Differences between GCMI and list-based approaches should be more cautiously considered when monitoring chronic conditions in the population. These approaches should be viewed as complementary rather than contradictory or interchangeable.

## Introduction

Developed in the early 2000s by the Euro-REVES group to monitor health expectancies and disability processes in Europe, the Minimum European Health Module (MEHM) is a set of three global questions on three different health dimensions: self-perceived health (SPH), global chronic morbidity and global activity limitation (GALI).^1^ The MEHM was intended to be used in various health and non-health surveys as a benchmark measure. For health surveys, it was recommended to be supplemented with the reporting of specific individual health conditions, limitations and activities restrictions.^2^ However, the MEHM is increasingly used in Europe and elsewhere as a self-sufficient set of questions to measure perceived health, chronic morbidity and disability. Unlike the GALI and, to a lesser extent, the SPH indicator, the metrological characteristics of the global chronic morbidity indicator (GCMI) have seldom been investigated. Based on a question (‘Do you have any longstanding illness or health problem?’) that was developed based on important conceptual work,^1^^,^^2^ the indicator has only been tested for reliability with Kappa = 0.77 in a test-retest over 20 days.^3^ Using a different global question that aggregates morbidity and handicap (‘Do you have a longstanding disease, condition or handicap?’), Van der Heyden et al. obtained a Kappa = 0.56 (over a year) and sensitivity ranging from 50% to 87% depending on the specific type of reported disease taken as the reference standard (*N* = 10 specific diseases).^4^ In the latter study in which two dimensions were aggregated, the sensitivity of the GCMI was much higher in subjects with poor health status, although the effect of multimorbidity (and the non-random association of chronic diseases) was not taken into account. These results might seem disturbing given that the GCMI is currently used to assess the burden of chronic morbidity and even compute life expectancy without chronic morbidity (i.e. morbidity-free life expectancy) in Europe,^5^ notably using data from European Union Statistics of Income and Living Conditions (EU-SILC) surveys.^6^

There is thus an urgent need to further document the metrological performance of the GCMI and to specially identify which aspects of chronic disease are captured or missed when the GCMI is used alone.

The main objectives of this study are (i) to further document the metrological performance (criterion and predictive validity) of the GCMI in assessing the burden of chronic morbidity in the general population and (ii) to examine the consequences of using the GCMI alone in terms of prevalence estimates and demographic and socioeconomic gradients compared with those obtained using list-based indicators of reported individual chronic conditions as used in many countries.^7^^,^^8^

## Methods

### Survey design and study population

We used data from two large nationwide representative surveys recently conducted in France using the MEHM and similar large lists of self-reported chronic conditions: (i) the Health, Health Care, and Insurance Survey from 2010 to 2014 (Enquête Santé et Protection Sociale, ESPS), which is a longitudinal health survey representative of individuals living in households in France in 2010^9^ and (ii) the 2021 edition of Santé publique France Health Barometer (SpF Barometer), which is a repeated cross-sectional telephone survey based on the random digit dialling of landline and mobile telephone numbers of individuals living in metropolitan France.^10^ Participation rates were considered satisfactory given the time and context (65% in ESPS and 44% in SpF Barometer), leading to the inclusion of 9815 participants aged ≥25 years in ESPS study and 3567 participants aged 65–84 years in SpF Barometer who were assessed with the MEHM and interviewed regarding the presence of individual chronic conditions.

### Minimum European Health Module and recorded chronic conditions and sociodemographic indicators

The MEHM and its three items (SPH, GALI and GCMI) were worded as recommended by the Euro-REVES group^1^ in both surveys. Based on earlier findings,^11^ this study considered very similar lists of chronic or recurrent conditions occurring within the past year that were consistently associated with having an independent impact on health status indicators (activity limitations, perceived health, mortality) across different surveys and/or within surveys across different indicators. Self-reported chronic conditions (*N* = 35, ESPS survey and *N* = 30, SpF Barometer survey, shown in Supplementary table S1) were recorded independently of the MEHM in separate parts of the questionnaires. Sociodemographic indicators included age (years), gender (male, female), marital status (married, living with a partner, separated, divorced, widowed and single), education level (less than secondary, secondary and tertiary according to ISCED codes 0–2, 3 and 4–8), employment status (paid employment, unemployed, retired, other), occupation (manager or professional, middle manager or teacher, manual and other worker, no occupation and student), income (categorized into terciles when provided, plus a category ‘not provided’).

### Outcomes

The health status indicators of interest in the longitudinal ESPS study included mortality (but not causes of death) and score changes in terms of functioning and perceived health measures when repeated measurements were available (i.e. GALI, SRH). Slightly more than half of participants from the ESPS 2010 sample (*N* = 5424, one person per household) were followed up and reassessed in 2014 to ascertain their vital and health status.

### Statistical analysis

Criterion validity was assessed against independently reported individual chronic conditions. Sensitivity (%) of the GCMI was computed by the number and type of common individual chronic conditions reported in the ESPS and SpF Barometer surveys. To address possible contamination biases due to multimorbid associations, analysis according to the type of chronic condition was stratified by multimorbid status (no/yes). Sensitivity (%) of the GCMI was also computed regarding the presence or absence of any chronic condition recorded in the considered lists (*N* = 35 and *N* = 30 for the ESPS and SpF Barometer surveys, respectively) as well as in the lists used for the purpose of chronic disease surveillance in the Dutch^7^ (*N* = 10) and BRFSS^8^ (*N* = 7) subsets (lists given in Supplementary table S1). Specificity (%) of the GCMI was computed for the absence of any chronic conditions in these lists.

Sociodemographic predictors of the GCMI and specific chronic conditions associated with it were further investigated using multivariate logistic regression models. Multivariate models for mortality and deteriorated health over 4 years in the longitudinal ESPS study were also developed using logistic regression to assess and compare the predictive ability of the GGCI with those of specific chronic conditions included in the aforementioned lists.

Finally, demographic and socioeconomic gradients (age, gender, education, occupation and income) were produced using the GCMI and alternative indicators based on the lists of reported individual chronic conditions (Dutch^7^ and BRFSS^8^ subsets).

All the analyses were performed separately for the two surveys using appropriate weights to provide valid estimates for the French population (2010 for ESPS and 2021 for SpF Barometer), while taking into account the unequal probabilities of selection resulting from the sample design, non-response and non-coverage in both surveys. SAS version 9.2 software (SAS Institute, Cary, NC) was used.

## Results

### Subject characteristics and measures of chronic conditions

The main characteristics of the two survey samples are presented in Supplementary table S1. The GCMI was positively endorsed (‘yes’) by 39.5% and 58.6% of ESPS and SpF Barometer participants, respectively, with 67.6% and 89.5% of these participants reporting at least one chronic condition among the list of individual chronic conditions. When considering only participants with the same age interval (65–84 years), similar levels of GCMI and chronic conditions were reported in both surveys. Supplementary figure S1 shows the percentages of positive endorsement of these two indicators according to gender, age group and survey. In the ESPS, the percentages of subjects reporting at least one chronic condition from the list of individual conditions were systematically higher than those of subjects endorsing the GCMI in all age groups. Women consistently reported higher levels of chronic condition(s) than men, although the differences decreased as age increased. When considering the same age group (65–84 years), results were very similar in both surveys: a higher percentage of subjects reported at least one chronic condition in the list compared with those endorsing the GCMI, with minimal differences between men and women.

### Sensitivity and specificity of the GCMI

In subjects reporting only one chronic condition, the sensitivity of the GCMI varied substantially depending on the types of chronic conditions: from 20% or less (obesity, migraine, inflammatory bowel diseases, peptic ulcer, anxiety, eye and ear impairments) to around 80% (diabetes, epilepsy, injury sequelae) (table 1). Sensitivity did not consistently exceed 50%, even for cancers and cardiovascular disorders. Sensitivity of the GCMI was higher and often much higher for all types of individual conditions in multimorbid subjects. Overall, sensitivity values were higher and sometimes much higher in the SpF Barometer survey (e.g. cancer, obesity, anxiety). By contrast, very similar values of sensitivity were observed in both surveys with an increasing number of conditions reported in multimorbid subjects. The threshold of 80% sensitivity of the GCMI was not reached until five concomitant conditions (table 1). Compared with the indicators based on reported individual chronic conditions in standard lists of varying sizes (*N* = 7–35), the sensitivity of the GCMI ranged from 53% to 67% in the ESPS and from 48% to 58% in the SpF Barometer, while the specificity ranged from 78% to 90% in the ESPS and from 79% to 91% in the SpF Barometer (table 2). The 10% ‘false positive’ subjects who endorsed the GCMI without reporting any of the listed individual chronic conditions did not have a consistent profile except from being rather healthy in both surveys (data not shown).

**Table 1:** Sensitivity (%) of the GCMI by case (monomorbid/multimorbid) and number of individual chronic conditions reported in the 2010–2014 ESPS and 2021 SpF Barometer surveys, France

	ESPS survey, ≥25 years		SpF Barometer survey, 65–84 years	
Type of individual chronic condition reported ^a^	Monomorbid subjects	Multimorbid subjects	Monomorbid subjects	Multimorbid subjects
Hypertension	46	74	39	79
Ischaemic heart disease or myocardial infarction	86	90	–	–
Cardiac rhythm disorders	50	82	45	84
Cancer (any except for skin)	50	79	85	86
Chronic obstructive pulmonary disease	40	80	–	–
Asthma	57	80	–	–
Migraine	17	57	–	–
Epilepsy	81	83	–	–
Depression	44	70	–	–
Anxiety	18	65	29	72
Ear ailments with hearing impairment	26	70	20	67
Eye ailments with uncorrected vision loss	20	72	31	75
Peptic ulcer	19	66	–	–
Inflammatory bowel diseases	11	62	–	–
Thyroid disorders	52	77	47	76
Diabetes	78	90	86	88
Obesity (BMI >30)	9	70	32	73
Lower back pain	27	65	28	69
Osteoarthritis of peripheral joints	36	69	34	68
Osteoporosis	30	72	11	61
Injury sequelae	76	76	–	–
At least one chronic condition reported among the previous list	53		57	
Number of conditions reported in the list				
1	32		42	
2	52		53	
3	63		61	
4	73		70	
5	80		81	
6	87		82	
7	89		93	
8	92		93	
9	99		82	
10	100		97	

a At least 15 subjects with the condition.

**Table 2:** Sensitivity and specificity (% with 95% confidence interval) of the GCMI according to several reference indicators based on the reported individual chronic condition list from the 2010–2014 ESPS and 2021 SpF Barometer surveys, France

	ESPS survey		SpF Barometer survey	
	Sensitivity	Specificity	Sensitivity	Specificity
Complete set^a^	53 (52–55)	90 (89–91)	57 (55–59)	90 (89–91)
Dutch subset^b^	58 (57–60)	84 (83–85)	48 (46–50)	79 (78–81)
BRFSS subset^c^	67 (65–69)	78 (77–79)	53 (51–55)	85 (84–87)

a At least one reported in *N* = 35 (ESPS) or 30 (SPF Barometer); see Supplementary table S1.

b At least one reported in *N* = 10; see Supplementary table S1.

c At least one reported in *N* = 7; see Supplementary table S1.

### Factors associated with GCMI endorsement

Several sociodemographic variables were consistently associated with positive GCMI endorsement independently of the number of individual chronic conditions reported (Supplementary table S2). In the ESPS, associations were found for gender (13% fewer women than men), age (younger participants less than older participants with significantly higher risks for the age group 45–54 years vs. 25–34 years) and education (10% fewer participants with lower education than with more education). Similar but stronger associations were found with the SpF Barometer for gender (31% fewer women than men) and education (36% fewer participants with lower education than with more education). In both studies, a negative interaction was found between older age and lower education level on the one hand and the number of chronic conditions on the other: the propensity to endorse the GCMI increased less strongly with the number of conditions in older participants and in those with less education compared with the other age and education categories.

The type of chronic conditions reported was found to be strongly associated with GCMI endorsement independently of the sociodemographic variables and the number of conditions reported (Supplementary table S3). Very similar findings were found for the ESPS and SpF Barometer: cardiovascular diseases, hypertension, diabetes and cancers were associated with a higher endorsement, whereas obesity, low back pain, arthritis, osteoporosis and eye and ear ailments with a lower or much lower endorsement. However, some noteworthy differences were found with regard to the surveys for stroke, asthma and injury sequelae (positively associated with GCMI endorsement in the ESPS but not in the SpF Barometer) as well as anxiety (negatively associated with GCMI in the SpF Barometer but not in the ESPS).

### GCMI as a predictor of health outcomes

In the longitudinal ESPS study, the GCMI was found to be predictive of death [OR = 2.24, 95%CI (1.41–3.55)] and new activity limitations [OR = 2.33, 95%CI (1.75–3.10)], with the levels of risks (gender- and age-adjusted risks) being similar to those found with having at least one reported individual chronic condition in the three considered lists (table 3). However, though significant, the risk of new health deterioration was lower with the GCMI [OR = 1.77, 95%CI (1.30–2.41)] than that found with reporting at least one chronic condition from the ESPS list [OR = 3.73, 95%CI (2.60–5.36)] and the Dutch list [OR = 3.01, 95%CI (2.20–4.11)]. The GCMI remained predictive of death after adjusting for the number of reported chronic conditions.

**Table 3:** Global chronic condition indicator (GCMI) and individual chronic conditions in lists as predictors of 4-year mortality, new activity limitations^a^, new health deterioration^b^ in the 2010–2014 ESPS survey, France

Indicator	Mortality	New activity limitations	New health deterioration	Covariates adjusted for
GCMI	2.24 (1.41–3.55)	2.33 (1.75–3.10)	1.77 (1.30–2.41)	Age, sex
Any chronic condition in the complete set (*N* = 35)	2.17 (0.92–5.13)	3.33 (2.32–4.78)	3.73 (2.60–5.36)	Age, sex
Any chronic condition in the complete set without obesity	2.09 (0.93–4.67)	3.34 (2.36–4.72)	3.49 (2.47–4.93)	Age, sex
Any chronic condition in the Dutch subset	1.85 (0.98–3.49)	3.80 (2.77–5.22)	3.01 (2.20–4.11)	Age, sex
Any chronic condition in the complete BRFSS subset	2.02 (1.16–3.51)	2.91 (2.14–3.97)	2.36 (1.73–3.22)	Age, sex
GCMI	1.92 (1.17–3.15)	1.32 (0.93–1.86)	1.30 (0.92–1.84)	Age, sex and number of individual conditions endorsed

a Limitation, severe or not in 2014 in those not limited in 2010.

b Health rated less than good in 2014 in those having good/very good health in 2010.

Odds ratios and 95% confidence intervals are obtained by logistic regression.

### Prevalence of chronic conditions and demographic and socioeconomic gradients using the GCMI and other list-based indicators

As expected, the prevalence of all chronic disease indicators was lower in the ESPS than in the SpF Barometer, where the study population was much older. In the SpF Barometer, no sociodemographic or economic gradients were observed. In the ESPS, gradients for more chronic diseases in lower sociodemographic or economic groups were often strong, and the GCMI compared favourably with other indicators except for the Dutch subset, which produced the most distinct differences (table 4).

**Table 4:** Overall prevalence (%) of chronic conditions and demographic and socioeconomic gradients using the GCMI and list-based indicators of reported individual chronic conditions in the 2010–2014 ESPS and 2021 SpF Barometer surveys, France

	ESPS survey					SpF Barometer survey				
	GCMI endorsed	Any individual chronic conditions in the complete set (*N* = 35) endorsed	Any individual chronic condition in the complete set (without obesity) endorsed	Any individual chronic conditions in the Dutch subset endorsed	Any individual chronic conditions in the BRFSS subset endorsed	GCMI endorsed	Any individual chronic conditions in the complete set (*N *= 30) endorsed	Any individual chronic conditions in the complete set without obesity endorsed	Any individual chronic conditions in the Dutch subset endorsed	Any individual chronic conditions in the BRFSS subset endorsed
Overall prevalence of chronic condition	39.5	67.6	64.6	55.8	38.6	58.6	89.5	88.4	79.7	69.0
Gender										
Male	38	63	59	37	52	60	87	86	66	79
Female	41	72	69	40	59	58	91	90	71	80
Ratio of extreme values	1.07	1.14	1.17	1.06	1.13	0.97	1.05	1.04	1.07	1.01
Age										
25–34 years	21	43	39	7	30					
35–44 years	25	52	47	12	36					
45–54 years	34	65	62	32	52					
55–64 years	49	81	79	56	69					
65–74 years	61	90	89	73	82	57	88	87	67	78
75–84 years	64	95	94	83	89	61	92	91	72	82
≥85 years	66	96	96	87	91					
Ratio of extreme values	3.12	2.22	2.45	12.99	3.02	1.07	1.04	1.04	1.08	1.05
Education										
Less than secondary	51	80	78	58	70	58	91	90	72	82
Secondary	35	64	61	33	52	62	86	85	66	75
Tertiary	31	56	54	22	43	60	86	85	60	74
Ratio of extreme values	1.64	1.44	1.45	2.70	1.64	0.96	1.07	1.06	1.20	1.11
Occupation (present)										
Manager, professional	39	64	62	35	51					
Middle manager, teacher	39	67	64	35	55					
Retired	40	69	66	43	58					
No occupation or student	48	75	73	49	62					
Ratio of extreme values	1.24	1.18	1.18	1.41	1.21					
Household incomes										
Lower tercile	48	76	74	50	64	58	90	89	73	82
Middle tercile	37	66	62	36	53	60	91	90	71	82
Upper tercile	33	60	57	27	48	59	88	87	63	76
Not provided	40	69	67	43	59	56	86	84	67	76
Ratio of extreme values	1.44	1.28	1.30	1.84	1.35	0.97	1.03	1.02	1.16	1.07

## Discussion

This study based on two large representative general population samples with cross-sectional and longitudinal data on the MEHM and numerous chronic conditions allowed us to comprehensively evaluate the validity of the GCMI, to analyze the implications of its use in terms of prevalence and to determine the demographic and socioeconomic gradients of chronic conditions in the general population. The results, along with those concerning alternative indicators based on lists of specific chronic diseases, allow us to propose the effective and appropriate use of the GCMI and list-based indicators for the surveillance of chronic conditions.

### Lack of criterion validity with the GCMI

This study confirms that the GCMI lacks criterion validity and especially sensitivity to capture the presence of common chronic conditions, even in highly multimorbid subjects. The lack of sensitivity is substantial, as it overlooks about one in two chronic conditions. More importantly, it also varies depending on the type of condition and especially the socioeconomic status of the respondent. Individuals with obesity, impaired hearing or impaired vision as well as older people and those with a lower education level were less prone to endorse the GCMI, thus potentially inducing biased estimates of demographic and socioeconomic gradients. A previous study observed the lack of sensitivity in a question resembling the GCMI, although compared with our study, the investigation was less comprehensive and the results were less conclusive, notably in terms of sociodemographic predictors.^4^

Our results have obvious consequences for some of the current common uses of the GCMI. First, it does not seem appropriate to use the GCMI as a ‘filter’ to select subjects for the further exploration of chronic conditions or as an adjustment covariate to take into account the presence of chronic morbidity: the filter is not sensitive enough and the adjustment is imperfect, thereby producing biased estimates.^12^ Second, it is especially problematic to use the GCMI to produce estimates of chronic morbidity prevalence and chronic morbidity-free life expectancy^13^ (CMFLE or equivalent designations^14^) and to compare these indicators across countries, regions or populations as is currently done within the EU, for example, based on data from the EU-SILC surveys.^6^ The prevalence of chronic conditions is overly underestimated (and CMFLE overly overestimated) and the comparisons are biased by both non-differential errors (in favour of the null hypothesis) and confounding, since populations differ in terms of their demographic and socioeconomic composition, which is obvious in the case of the EU.

### Different perceptions of different chronic conditions

Our results show that some conditions were not commonly perceived as chronic diseases by individuals. Migraine, eye conditions, peptic ulcer disease, inflammatory bowel disease, obesity and even osteoporosis in the elderly were largely overlooked with the GCMI contrary to diabetes, injury sequelae and epilepsy, which were better captured. These differences can probably be explained by different levels of knowledge about what constitutes a chronic disease (e.g. low knowledge about obesity or peptic ulcer disease versus high knowledge about diabetes), the degree of impairment in daily life (e.g. high impact of epilepsy) and the life-threatening character of the disease (e.g. ischemic heart disease, cancer). These results further indicate that knowledge about highly underperceived chronic conditions such as obesity or peptic ulcer should probably be enhanced through health promotion or disease prevention initiatives to improve their management.

### Predictive ability of health events with the GCMI: an indicator of perceived chronic conditions

Another important result of this study is that despite its lack of criterion validity, the GCMI was able to predict health events independently of individual chronic conditions. The GCMI was, therefore, able to capture aspects relating to ‘prognosis’ in terms of limitations, altered perceived health and even mortality, which is in line with the finding that the GCMI tends to capture more severe and longstanding chronic conditions, as above shown. Similar predictive values, independent of the specific diseases mentioned by subjects, were found with perceived health or quality-of-life indicators in various contexts, for example.^15^^,^^16^ It would thus be pertinent to rename the GCMI as the ‘perceived chronic condition indicator’, which goes beyond the objective assigned to this question by the Euro-REVES group, namely to provide ‘broad information on the health status of a population and on the impact of chronic morbidity on social behaviours’.^17^

### Complementary uses of the perceived and list-based chronic condition indicators

In addition to highlighting the value and limitations of the GCMI, this study showed how strikingly different results were obtained when list-based approaches were used for the assessment of chronic conditions. Interestingly, the figures of prevalence and sociodemographic gradients for the GCMI were very close to those obtained with a list-based indicator based on the BRFSS short list (*N* = 7, mostly impacting mortality), but much more different from those obtained with indicators based on the Dutch subset (*N* = 10, more diverse in terms of impacts) and even more so from those obtained with larger and more complete subsets of chronic conditions as in our surveys (*N* ≥ 30, selected to have an impact on activity limitations, perceived health or mortality). As expected, mainly due to ceiling effects, the lower overall prevalence values of the GCMI were associated with steeper socioeconomic gradients. In view of these results, we recommend using the GCMI in a complementary way with measures based on lists of individual chronic conditions. Among these measures, which are predominantly used worldwide,^7^^,^^8^^,^^18–22^ those using large sets of chronic conditions are probably preferable. The creators of the MEHM and the GCMI recommended the complementary use of both types of indicators^2^ but this recommendation was largely ignored in implementation: one or the other approach tended to be used and sometimes one after the other.^23^

### Study strengths and limitations

This study has several strengths: (i) the use of cross-sectional and longitudinal data from two large and nationally representative surveys; (ii) a rich body of data with dozens of chronic conditions along with the MEHM and (iii) replicated analyses and consistent results obtained across samples and indicators, which provide robust evidence in response to the study questions. Several limitations should also be highlighted: (i) the reliance on self-reported data for chronic conditions; (ii) the limited consideration of multimorbidity, which has become a major issue in France^11^ and elsewhere^24^ (and would probably merit a multinomial approach as recently suggested^25^) and (iii) the limited power to detect small effects associated with less frequent conditions or even moderate effects in longitudinal analyses with rare events. In particular, we provide comparisons of the GCMI with self-reported chronic conditions, which are also subject to several biases: memory bias, bias associated with social desirability as in the case of obesity or mental health conditions and bias favouring more symptomatic conditions.^11^

### Implications

While chronic conditions or diseases have defied medical thinking for more than 2000 years,^26^ with new definitions of the phenomenon still being regularly proposed,^27^ determining the best way to monitor them in the population represents a daunting task. As for the indicators of other health dimensions used in health surveillance (disability,^28^^,^^29^ quality of life,^30^ mental health^31^), the ‘global’ or ‘self-reported’ assessment of chronic conditions based on individuals’ own perceptions seems to provide relevant and predictive information that merits consideration for population-based surveillance in complement to the list-based assessments of individual conditions, which, even if they are self-reported, are derived from medical assessments. This complementary use was indeed recommended by the Euro-REVES group.^2^ However, more research is needed on the perceptual assessment of chronic diseases, notably by testing alternative formulations and/or increasing the number of responses. This will allow us to define the conditions for its valid, unbiased and ethical use together with its complementary use with list-based assessments. Furthermore, there is also need to better standardize and harmonize these lists across similar countries. At the moment, however, the possibility of having divergent results should be regarded as a problem to be addressed rather than as an opportunity to choose the more convenient figure according to the pursued objectives, especially when computing life expectancy without chronic disease.

## Conclusion

This study showed that the GCMI lacks validity, especially sensitivity when it is used to measure chronic disease in the population. Furthermore, its use may produce biases when constructing demographic and socioeconomic gradients. Nevertheless, the GCMI predicts health events independently of individual conditions, especially mortality, and captures important, albeit perceptual, dimensions of the phenomenon of chronic disease, which deserves further consideration. However, differences in the prevalence and demographic and socioeconomic gradients obtained with the GCMI and list-based approaches should be taken into account, and both approaches should be seen as complementary rather than as contradictory or interchangeable.

## Supplementary Material

ckae064_Supplementary_Data

## Data Availability

The authors are not legally authorized to share or publicly publish the dataset. Requests for access to the data should be addressed to the French Institute for Research and Information in Health Economics (ESPS 2010 survey) or to Santé publique France (2021 Health Barometer).
