# Supplementary material for: Metrological performances of the global chronic morbidity indicator of the Minimum European Health Module and implications for chronic disease prevalence and socioeconomic gradient estimations
Source: Eur J Public Health. 2024 Mar 29;34(4):774–80. doi: 10.1093/eurpub/ckae064 (PMC11293826; doi:10.1093/eurpub/ckae064)
Supplement: ckae064_Supplementary_Data [file ckae064_supplementary_data.pdf]

Metrological performances of the global chronic morbidity indicator of the Minimum European Health Module and implications for chronic disease prevalence and socioeconomic gradient estimations

Supplementary material

Supplementary Table S1. Description of the studied samples (ESPS and SpF Barometer surveys, France 2010-14 and 2021).

Supplementary Figure S1. Frequency of positive endorsement of the GCMI and individual chronic conditions in the 2010-14 ESPS (n=35) and 2021 SpF Barometer (N=30) lists.

Supplementary Table S2. Independent predictors of GCMI in the 2010-14 ESPS and 2021 SpF Barometer surveys, France. Final main effects.

Supplementary Table S3. Assocations of the GCMI with individual chronic conditions in the 2010-14 ESPS and 2021 SpF Barometer surveys, France.

Supplementary Table S1. Description of the studied samples (ESPS and SpF Barometer surveys, France 2010-14 and 2021). All figures are weighted percentages unless otherwise indicated.

	ESPS Survey (France, 2010-4)		SpF Barometer Survey (Metropolitan France, 2021) (N=3,567)
	Whole sample (N=9,815)	65-84 yrs subsample (N=1,932)*	
Gender, female	53.9	54.0	54.6
Age			
25-34 years	18.3		
35-44 years	20.8		
45-54 years	18.6		
55-64 years	18.5		
65-74 years	12.4	57.8	63.1
75-84 years	9.1	42.2	36.9
≥ 85 years	2.3		
Education			
Less than secondary	33.4	62.7	67.7
Secondary	41.0	27.0	12.3
Tertiary	25.6	10.3	19.9
Marital status			
Married/living with a partner	69.5	63.2	67.6
Separated/divorced/widowed/single	30.5	36.8	32.4
Occupation (current)			
Manager, professional	17.2	–	–
Middle manager, teacher	38.9	–	–
Other, manual worker	41.1	–	–
No occupation or student	2.8	–	–
Employment status			
Paid employment	56.7	1.1	2.8
Unemployed	6.1	0.0	0.2
Retired	29.6	92.9	96.3
Other	7.7	5.9	0.7
Household income			
Lower tercile	28.8	44.1	28.7
Middle tercile	27.1	23.4	31.3
Upper tercile	28.8	13.6	27.8
Not provided	15.2	18.9	12.2
**Minimum European Health Module**			
Global Activity Limitation Indicator (GALI)			
Not limited	73.3	53.4	58.1
Limited, not severely	18.8	30.6	34.0
Limited, severely	7.9	16.0	7.9
Self-perceived health (SPH)			
Very good - Good	65.4	43.1	62.0
Fair	26.2	40.2	21.8
Bad - Very bad	8.3	16.7	16.2
Global chronic morbidity indicator (GCMI)			
Yes	39.5	62.4	58.6
No	60.5	37.6	41.4

* Descriptive statistics of this subsample are shown for comparison with Barometer Survey similar population

Supplementary Table S1 (continued). Description of the studied samples (ESPS and SpF Barometer surveys, France 2010-14 and 2021). All figures are weighted percentages unless otherwise indicated.

	ESPS Survey (France, 2010-4)		SpF Barometer Survey (Metropolitan France, 2021) (N=3,567)
	Whole sample (N=9,815)	65-84 yrs subsample (N=1,932)*	
Individual chronic conditions			
HIV infection	0.03	0.0	–
Hypertension	21.0	49.8	35.7
Ischemic heart disease or myocardial infarction	3.1	8.6	2.2
Valvulopathy	–	–	0.6
Stroke	1.7	4.6	1.8
Peripheral arterial disease	1.7	4.4	2.7
Cardiac rhythm disorders	5.8	14.1	14.1
Heart failure	0.6	1.7	0.1
Cancer (any except skin cancer)	4.8	11.4	5.7
Non-malignant blood disorder	–		0.8
Chronic obstructive pulmonary disease (COPD)	3.0	6.3	5.9
Asthma	4.8	3.9	5.3
Migraine	9.3	5.8	–
Epilepsy	1.0	1.3	–
Alzheimer's disease and other dementias	0.3	1.2	1.9
Parkinson's disease	0.4	1.1	0.4
Multiple sclerosis	0.2	0.2	–
Depression	7.6	10.6	6.5
Anxiety	12.7	17.1	24.0
Schizophrenia	0.1	0.1	–
Substance abuse	0.3	0.1	–
Ear ailments with deafness	11.3	24.1	28.6
Eye ailments with uncorrected vision loss	7.4	21.0	11.5
Peptic ulcer	2.4	4.1	–
Inflammatory bowel diseases	3.7	2.7	0.2
Chronic liver diseases	0.9	1.1	0.2
Thyroid disorders	6.4	10.3	11.0
Diabetes	6.3	15.7	18.1
Obesity (BMI >30)	13.6	19.3	20.0
Rheumatoid arthritis	0.5	0.8	0.8
Other (non-rheumatoid) inflammatory arthritis	1.1	2.6	0.1
Other inflammatory condition	–		2.3
Lower back pain	18.7	24.2	43.6
Osteoarthritis of peripheral joints	20.4	42.6	39.7
Osteoporosis	4.2	10.9	10.1
Kidney failure	0.2	0.5	5.0
Urinary incontinence	0.4	0.5	–
Injury sequelae	1.8	2.5	9.3
Number of individual chronic conditions from the preceding list			
0	32.4	7.8	10.5
1	24.3	15.1	16.7
2	16.2	21.0	18.3
3	10.4	17.1	15.1
4	6.6	12.5	12.9
≥5	10.1	26.6	26.6
At least one condition among the preceding list	67.6	92.2	89.5
At least one chronic condition from the Dutch subset list**	55.8	84.5	79.7
At least one chronic condition from the BRFSS subset list**	38.6	77.1	69.0
Followed-up***	96.9	95.2	–
Deceased 2010-2014	2.1	4.9	–
New GALI limitation after 4 years	16.6	32.0	–
New SPH deterioration after 4 years	16.9	33.6	–

* Descriptive statistics of this subsample are shown for comparison with Barometer Survey similar population

** The Dutch subset includes asthma or COPD, heart condition (ischemic heart disease or myocardial infarction, heart failure), stroke, hypertension, disturbance of stomach-intestines (peptic ulcer or Inflammatory bowel diseases; diabetes; low back pain, rheumatic or joint disorder (rheumatoid arthritis, other non-rheumatoid inflammatory arthritis, osteoarthritis of peripheral joints), migraine and cancer; and the BRFSS includes osteoarticular disorders (rheumatoid arthritis, other non-rheumatoid inflammatory arthritis, other inflammatory condition, osteoarthritis of peripheral joints), cancers, cardiovascular disease (Ischemic heart disease or myocardial infarction, heart failure), hypertension, kidney disease,COPD and diabetes.

*** The follow-up was scheduled in 5,424 subjects

Supplementary Figure S1. Frequency of positive endorsement of the GCMI and individual chronic conditions in the 2010-14 ESPS (n=35) and 2021 SpF Barometer (N=30) lists.

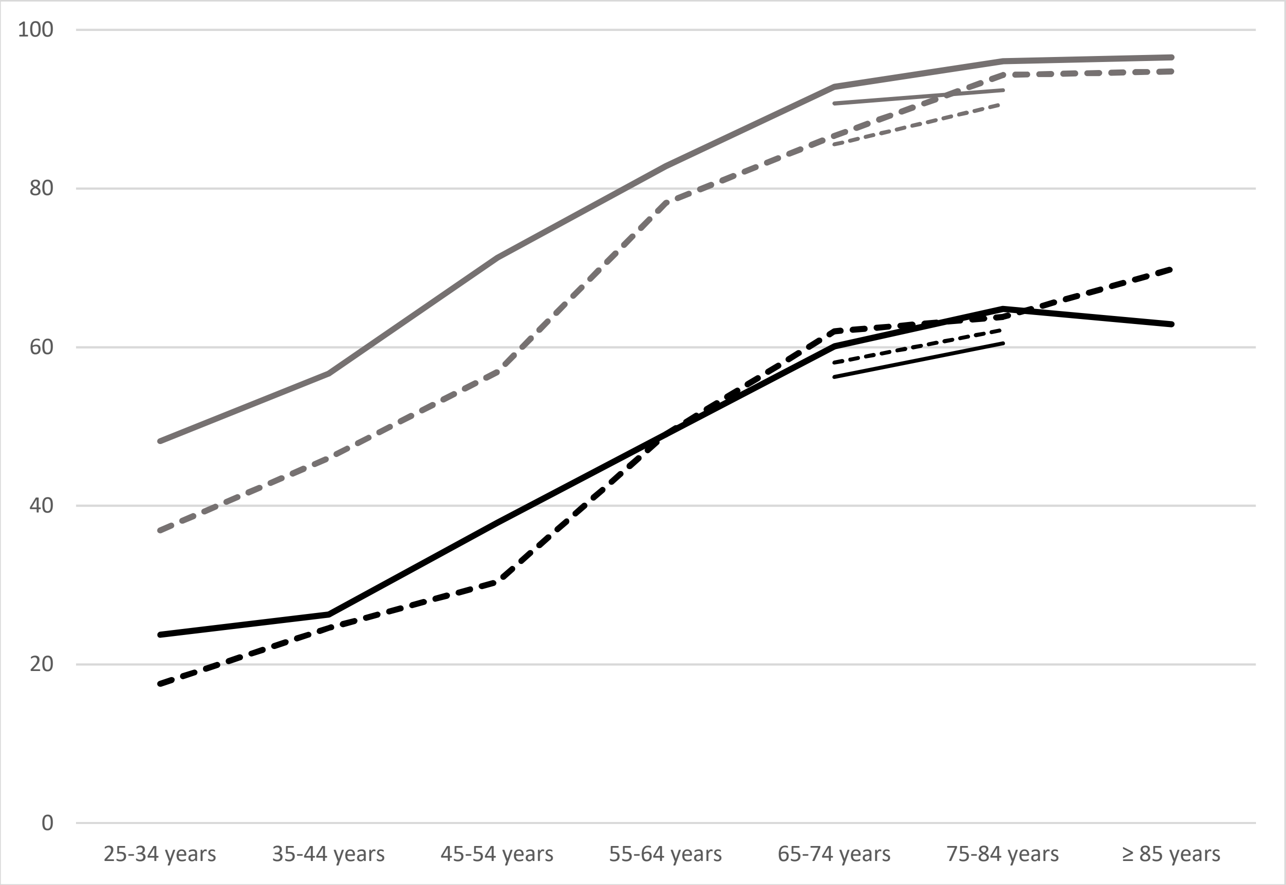

Women : full line, men dotted line ; ESPS survey : thick line, BSPF survey : thin line ; GCMI : black, in list : grey.

Supplementary Table S2. Independent predictors of GCMI in the 2010-14 ESPS and 2021 SpF Barometer surveys, France. Final main effects. Odds ratios and 95% confidence intervals.

	ESPS Survey	SpF Barometer Survey
Gender, female	0.87 (0.78-0.97)	0.69 (0.56-0.85)
Age		
25-34 years	1.00 (Ref)	
35-44 years	1.13 (0.94-1.36	
45-54 years	1.27 (1.06-1.53)	
55-64 years	1.70 (1.41-2.05)	
65-74 years	1.92 (1.54-2.40)	1.00 (Ref)
75-84 years	1.49 (1.14-1.94)	1.10 (0.90-1.36)
≥ 85 years	1.47 (0.97-2.21)	
Education		
Less than secondary	0.90 (0.77-1.04)	0.64 (0.51-0.79)
Secondary	0.82 (0.72-0.94)	0.93 (0.71-1.20)
Tertiary	1.00 (Ref)	1.00 (Ref)
Number of chronic conditions reported	1.94 (1.86-2.03)	1.65 (1,53-1,78)

Interactions with the number of conditions reported		
Gender, female	neg. P=0.04	NS
Age		
25-34 years	–	
35-44 years	NS	
45-54 years	NS	
55-64 years	NS	
65-74 years	neg. P=0.04	–
75-84 years	neg. P=0<0.0001	neg. P=0.03
≥ 85 years	NS	
Education		
Less than secondary	neg. P=0.0003	neg. P= 0.0004
Secondary	NS	NS
Tertiary	–	–

Supplementary Table S3. Assocations of the GCMI with individual chronic conditions in the 2010-14 ESPS and 2021 SpF Barometer surveys, France. Adjusted for age, gender, education level and number of conditions reported.

	ESPS Survey	SpF Barometer Survey
HIV infection	-	–
Hypertension	1.42 (1.22-1.66)	1.68 (1.31-2.15)
Ischemic heart disease or myocardial infarction	3.22 (2.06-5.04)	2.54 (1.12-5.80)
Stroke	2.14 (1.23-3.73)	0.93 (0.26-3.27)
Peripheral arterial disease	0.96 (0.54-1.73)	1.04 (0.45-2.39)
Cardiac rhythm disorders	1.59 (1.21-2.09)	1.40 (0.88-2.22)
Heart failure	1.44 (0.50-4.18)	–
Cancer (any except for skin)	1.65 (1.24-2.19)	2.72 (1.34-5.52)
Chronic obstructive pulmonary disease	1.14 (0.79-1.67)	1.28 (0.61-2.67)
Asthma	2.86 (2.14-3.82)	0.67 (0.31-1.45)
Migraine	0.52 (0.42-0.64)	–
Epilepsy	3.16 (1.47-6.78)	–
Alzheimer's disease and other dementias	2.41 (0.71-8.12)	–
Parkinson's disease	2.13 (0.70-6.46)	–
Multiple sclerosis	–	–
Depression	0.84 (0.66-1.08)	0.68 (0.36-1.27)
Anxiety	2.41 (0.71-8.12)	0.60 (0.44-0.80)
Schizophrenia	–	–
Substance abuse	1.14 (0.34-3.78)	–
Ear ailments with deafness	0.69 (0.57-0.84)	0.45 (0.32-0.61)
Eye ailments with uncorrected vision loss	0.67 (0.52-0.85)	0.86 (0.59-1.24)
Peptic ulcer	0.57 (0.39-0.83)	–
Inflammatory bowel diseases	0.58 (0.43-0.77)	–
Chronic liver diseases	2.97 (1.38-6.40)	1.36 (0.23-7.90)
Thyroid disorders	1.75 (1.35-2.26)	1.22 (0.80-1.86)
Diabetes	4.48 (3.25-6.18)	3.75 (2.37-5.93)
Obesity (BMI >30)	0.77 (0.65-0.90)	0.89 (0.64-1.24)
Rheumatoid arthritis	13.21 (3.36-51.90)	–
Other (non-rheumatoid) inflammatory arthritis	3.06 (1.50-6.21)	–
Lower back pain	0.71 (0.60-0.83)	0.57 (0.43-0.76)
Osteoarthritis of peripheral joints	0.77 (0.66-0.91)	0.53 (0.39-0.73)
Osteoporosis	0.73 (0.53-0.99)	0.39 (0.25-0.60)
Kidney failure	–	1.07 (0.45-2.59)
Urinary incontinence	0.87 (0.32-2.42)	–
Injury sequelae	1.91 (1.16-3.15)	0.66 (0.39-1.13)
